# Editorial: Wood Development and Physiology in a Changing Climate

**DOI:** 10.3389/fpls.2022.906736

**Published:** 2022-04-21

**Authors:** Guohua Chai, Mengzhu Lu, Xiaohan Yang, Taku Demura, Wei Li, Quanzi Li

**Affiliations:** ^1^College of Resources and Environment, Qingdao Agricultural University, Qingdao, China; ^2^State Key Laboratory of Subtropical Silviculture, School of Forestry and Biotechnology, Zhejiang A&F University, Hangzhou, China; ^3^Biosciences Division, Oak Ridge National Laboratory, Oak Ridge, TN, United States; ^4^The Center for Bioenergy Innovation, Oak Ridge National Laboratory, Oak Ridge, TN, United States; ^5^Division of Biological Science, Graduate School of Science and Technology, Nara Institute of Science and Technology, Nara, Japan; ^6^State Key Laboratory of Tree Genetics and Breeding, Northeast Forestry University, Harbin, China; ^7^State Key Laboratory of Tree Genetics and Breeding, Chinese Academy of Forestry, Beijing, China

**Keywords:** tree, wood formation, physiology, biotechnology, metabolism

Wood of forest trees is a renewable, sustainable and easily workable material and has been widely used in construction, paper making, furniture, and as a feedstock for biofuels. Wood formation is the result of a highly ordered and finely regulated process. Secondary xylem (wood) and phloem are the inner and outer derivative products of the vascular cambium, respectively. Xylem is mainly composed of dead cells with thickened cell walls rich in cellulose, hemicelluloses, and lignin, which are responsible for providing mechanical support and conducting water and minerals for the trees. Phloem transports photoassimilates and signaling molecules, including phytohormones and peptides. Wood structure varies greatly between, and within, tree species. A thorough understanding of developmental processes and metabolic pathways during wood formation would provide a basis for improving wood biomass and modifying properties in forest trees.

Genome sequences of fast-growing tree species, such as *Populus trichocarpa* (Tuskan et al., 2006), *Eucalyptus grandis* (Myburg et al., 2014), *Pinus taeda* (Neale et al., 2014), *Paulownia fortunei* (Cao et al., 2021), *Betula platyphylla* (Chen et al., 2021), and *P. tabuliformis* (Niu et al., 2022), facilitate basic scientific research and motivate the development of biotechnological toolkits for functional genomics. Recently, multi-omics integration has been applied in studying the complexity of wood biology. Through transcriptomic, proteomic, fluxomic and phenomic analyses, Wang et al. (2018) estimated how changes in the expression of wood pathway genes could affect protein abundance, metabolic-flux, metabolite contents, and 25 wood traits, including tree-growth, density, strength, lignin and saccharification. A hierarchical transcriptional regulatory network (TRN), in which the transcription factor (TF) PtrSND1-B1, a master regulator of secondary cell wall (SCW) formation, directs 57 TF-DNA interactions through 17 TFs that transregulate 27 cell wall metabolic genes, was constructed based on quantitative transcriptomics and chromatin binding data (Chen et al., 2019). Despite great progresses in the field of wood biology, a lot of questions remain to be answered on the regulation of wood formation in trees.

This Frontiers Research Topic collected recent advances made through a combination of approaches of genetics and chemistry and improved new insights into the mechanisms of wood biology (anatomy, structure, and function).

This volume is organized in four sections: (1) Growth and Development, (2) Omics Analysis, (3) Gene Discovery, and (4) New Technologies and Resources.

## Growth and Development

Wood formation is initiated from the vascular cambium, and encompasses a series of developmental processes, including cambial division, xylem differentiation, SCW thickening and programmed cell death (PCD). Understanding the mechanisms underlying these processes is essential for optimizing molecular breeding of high-yield trees. Wang D. et al. reviewed the recent discoveries on the regulation of vascular cambium development by multiple internal signals and their crosstalks in trees. This study revealed that there exists a similar but more complex regulatory network orchestrating vascular cambium development in poplar in comparison with Arabidopsis. Jiang et al. summarized the progress in the studies on the transcriptional regulation and signaling of developmental programmed cell death (dPCD) during vegetative and reproductive development. Future perspectives on how to take full advantage of dPCD in plants were discussed, with the goal of improving the efficiency of wood biomass production.

## Omics Analysis

High-throughput sequencing and multi-omics analysis in tree species provide resources for studying the genetic basis of wood formation. Luo et al. compared the regulatory network of wood formation between juvenile wood (JW) and mature wood (MW) forming tissues using RNA sequencing (RNA-Seq) and whole genome bisulfite sequencing (WGBS), and revealed significant differences in transcriptional programs and patterns of DNA methylation between JW and MW. Li et al. employed RNA-Seq and isoform sequencing (Iso-seq) technologies to explore the gene expression profile across wood-forming tissues in the angiosperm *P. alba* × *P. glandulosa* and the gymnosperm *Larix kaempferi* (Lamb.) Carr. Differential mechanisms of wood formation, were identified at the transcriptional and post-transcriptional levels between angiosperm and gymnosperm tree species. Xiao Y. et al. combined transcriptomics, proteomics, and confocal Raman imaging techniques to analyze tension wood (TW), opposite wood (OW), and normal wood (NW) in *Catalpa bungei*, resulting in the identification of several key candidate genes that regulate lignin biosynthesis.

## Gene Discovery

Currently, more and more efforts are being made to identify and characterize the genes involved in wood formation for genetic engineering in trees. Six research articles in this Research Topic determined the role of specific genes controlling wood biomass formation and properties. Liu et al. over-expressed the small guanosine triphosphate binding protein gene *PagRabE1b* in poplar and characterized its function in SCW thickening and PCD. Chu et al. showed that the knockdown of poplar microRNA393 (miR393) using the short tandem target mimic (STTM) approach could improve the tree growth and thus improved biomass production. Xiao L. et al. performed an association study as well as epistasis and mendelian randomization (MR) analyses, and revealed the impact of the genetic interaction between PtomiR403b and its target *PtoGT31B-1* on wood formation. During the secondary growth, trees are able to modify the anatomic structure of the wood in response to environmental stresses. Rodriguez-Zaccaro et al. illustrated that the genetic architecture of vessel traits affecting hydraulic physiology and resilience to water stress is under genetic regulation, which is not simply influenced by tree height.

Wood cell walls are composite materials containing cellulose crystals and a tri-component interstitial gel, which is composed of water, hemicelluloses, and lignin. The lignin in dicotyledonous angiosperms is typically polymerized from three monolignol precursors, coniferyl alcohol, sinapyl alcohol, and *p*-coumaroyl alcohol, resulting in guaiacyl (G), syringyl (S), and hydroxyphenyl (H) subunits, respectively (Zhao, 2016). Engineering of lignin quantity and composition is important for reducing biomass recalcitrance. Wang Q. et al. found that the poplar gene *CONIFERALDEHYDE 5-HYDROXYLASE* (*CAld5H2*) was negatively regulated by the BEL1-like homeodomain (BLH) protein PagBLH6a that functions through a combinatorial regulation with multiple TFs for S monolignol biosynthesis. Su et al. revealed that down-regulation of *p-Hydroxycinnamoyl-Coenzyme A: Quinate/Shikimate p-Hydroxycinnamoyltransferase* (*HCT*) expression in poplar changed the contents and micro-distributions of wood cell wall components as well as the morphological characteristics of cells.

## New Technologies and Resources

The CRISPR/Cas systems are facile, highly efficient and widely used in diverse cells and organisms (Wada et al., 2022). Woody plants with long-life spans and outcrossing mating systems are difficult subjects for traditional mutagenesis methods. An et al. developed a new mannopine synthase (MAS)-CRISPR/Cas9 system in poplar, which can be used to simultaneously edit multiple genes with higher mutation rates. Mining and establishment of wood-associated databases are helpful for understanding the development and structure of wood. In this issue, two contributions focus on the database of wood formation regulation. Zhuang et al. developed a high-throughput screening system with a library of 517 wood-associated TFs sampled from *P. alba* × *P. glandulosa* cv 84K. Multiple regulatory modules of lignin biosynthesis during wood development were identified by screening this TF library using yeast-one hybrid (Y1H) and yeast-two-hybrid (Y2H) methods. Wang H. et al. established a *P. trichocarpa* stem differentiating xylem (PSDX) database, which integrates 144 RNA-Seq, 33 ChIP-seq, and six single-molecule real-time (SMRT) Iso-seq libraries and presents comprehensive measurements of gene expression and post-transcriptional regulation. Beside these wood-associated databases, Gupta et al. described the economically feasible wood biopreservation platform in *Lannea coromandelica* (Houtt.) Merr. against wood rotting fungi using extracts from weeds viz. *Lantana camara* L. and *Ageratum conyzoides* L. The use of this plant-based, environmentally sustainable preservative has tremendous potential for wood protection.

Biotechnology has rapidly advanced the practical application of molecular genetics in tree breeding. The articles in this volume provide an excellent update on wood biology research.

## Author Contributions

All authors participated either in the writing or editing of the editorial. All authors contributed to the article and approved the submitted version.

## Funding

Financial support for this work was obtained from National Natural Science Foundation of China (31972955 and 32101549), the Central Guidance on Local Science and Technology Development Fund of Shandong Province (YDZX2021112) and the Taishan Scholar Program of Shandong (tsqn202103092).

## Conflict of Interest

The authors declare that the research was conducted in the absence of any commercial or financial relationships that could be construed as a potential conflict of interest.

## Publisher's Note

All claims expressed in this article are solely those of the authors and do not necessarily represent those of their affiliated organizations, or those of the publisher, the editors and the reviewers. Any product that may be evaluated in this article, or claim that may be made by its manufacturer, is not guaranteed or endorsed by the publisher.
